# Nuclear Genome Assembly of the Microalga Nannochloropsis salina CCMP1776

**DOI:** 10.1128/MRA.00750-19

**Published:** 2019-10-31

**Authors:** J. A. Ohan, B. T. Hovde, X. L. Zhang, K. W. Davenport, O. Chertkov, C. Han, S. N. Twary, S. R. Starkenburg

**Affiliations:** a Bioscience Division, Los Alamos National Laboratory, Los Alamos, New Mexico, USA; Vanderbilt University

## Abstract

Nannochloropsis salina is a halotolerant, high-lipid-producing microalga that is being explored as a biofuel production species. Here, we report an improved high-quality draft assembly and annotation for the nuclear genome of *N. salina* strain CCMP1776.

## ANNOUNCEMENT

*Nannochloropsis* is a genus of eukaryotic microalgae (1) known for high lipid content and the ability to be maintained in large-volume outdoor cultures (2, 3). It can also produce auxiliary products such as the pigments astaxanthin, zeaxanthin, and canthaxanthin and the dietary supplement eicosapentaenoic acid (EPA) (4–7), an omega-3 fatty acid. Further, *Nannochloropsis* is tractable for genetic modification (8, 9), with evidence for homologous recombination in some strains (8). Nannochloropsis salina is a halotolerant strain known to accumulate 50 to 70% of its dry weight as lipid under nitrogen starvation (5, 10), making it an attractive candidate as a biofuel feedstock.

*Nannochloropsis salina* strain CCMP1776 was initially isolated in 1965 from Skate Point, Scotland (55.75°N, 4.96°W), and was deposited in the Bigelow culture collection in 1997.

CCMP1776 was cultivated in f/2 medium at room temperature under ∼50 microeinsteins per meter squared per second and a 24-h light regime. Cultures growing under linear growth were harvested using centrifugation. Cells were lysed in AP1 buffer with a single pass through an Avestin Emulsiflex B-15 press at 30,000 lb/in^2^. Genomic DNA was purified using the Qiagen DNeasy plant maxikit following the manufacturer’s protocols.

Genomic DNA from CCMP1776 was sequenced and assembled using a combination of Illumina (11) and 454 (12) technologies. For this genome, we constructed and sequenced an Illumina GAII shotgun library, which generated 466 million reads totaling 85 Gb (90× coverage), and 2 paired-end 454 Titanium libraries with an average insert size of 5 kb, which generated 2,786,633 reads totaling 7.1 Gb of 454 data (16× coverage). The 454 Titanium standard data and the 454 paired-end data were assembled together with Newbler version 2.3 (091027_1459). The Newbler consensus sequences were computationally shredded into 10-kb overlapping fake reads (shreds) using an in-house script, resulting in 1.5× coverage of this assembly. Illumina sequencing data were assembled with Velvet version 1.0.13 (13), and the consensus sequence was computationally shredded into 10-kb overlapping shreds. We integrated the 454 Newbler consensus shreds, the Illumina Velvet consensus shreds, and the read pairs in the 454 paired-end library using parallel Phrap version 1.080812 (High Performance Software, LLC). Possible misassemblies were corrected using Gap Resolution (14) and Dupfinisher (15). The Gap Resolution software is available from the Department of Energy and the Lawrence Berkeley National Laboratory.

The final genome assembly was 27.6 Mbp contained in 194 scaffolds. The *N*_50_ value of this assembly is 828,788 bp, and the GC content is 54.88%. Genome annotation was performed using the BRAKER version 2 training and annotation pipeline (16) utilizing 254 million transcriptomic reads (paired end, 2 × 150 bp). Functional annotation of the 10,522 genes was performed using InterProScan 5 (17) and BLASTp searches against the UniProt (18) protein BLAST database. This genome will spur the continued development of algae for use as biofuel feedstock and provide prerequisite information needed for genetic manipulation.

### Data availability.

All sequences have been deposited in NCBI under BioSample number SAMN10354914 and GenBank accession number SDOX00000000. Genome assembly and annotations are also available at greenhouse.lanl.gov. The 454 raw sequencing data are available under NCBI SRA numbers SRR9992831 and SRR9992828. The Illumina raw reads are available under NCBI SRA number SRR9992830. The transcriptomic reads have been deposited under NCBI SRA number SRR9992829.
